# Electronic Systems for Monitoring Pediatric Gait Biomechanical Parameters: A Systematic Review of Embedded Technologies and Human–Machine Interfaces

**DOI:** 10.3390/s26103164

**Published:** 2026-05-16

**Authors:** Omar Freddy Chamorro-Atalaya

**Affiliations:** Facultad de Ingeniería Mecánica y Electrónica, Universidad Nacional Tecnológica de Lima Sur, Lima 15834, Peru; ochamorro@untels.edu.pe

**Keywords:** pediatric gait biomechanics, electronic monitoring systems, wearable sensors, plantar pressure, inertial measurement units, embedded technologies

## Abstract

**Highlights:**

**What are the main findings?**

- Wearable electronic systems and inertial measurement unit (IMU)-based sensing predominate in pediatric gait monitoring, reflecting a shift toward portable, ecologically valid assessment.
- Most systems focus on motion-derived outcomes, whereas HMI modalities are rarely described, and region-specific plantar-load distribution is infrequently assessed.

**What are the implications of the main findings?**

- The limited incorporation of interaction modalities suggests that current systems still provide restricted support for personalized feedback and rehabilitation in pediatric settings.
- Future pediatric gait technologies may benefit from integrating multimodal sensing, zonal plantar-load assessment, and user-centered interaction mechanisms.

**Abstract:**

Electronic systems are increasingly used to support pediatric gait assessment by enabling objective measurement of biomechanical parameters beyond traditional laboratory settings. However, although technological development has expanded in adult populations, the extent to which embedded technologies and human–machine interaction (HMI) modalities have been integrated into pediatric monitoring systems remains unclear. This systematic review synthesizes evidence published between 2015 and 2025 on electronic systems applied to pediatric gait biomechanics. The review followed PRISMA guidelines, was registered in PROSPERO (CRD420251230372), and adopted a descriptive synthesis approach. A total of 2619 records were identified, and after eligibility assessment and methodological quality appraisal using CASP, 34 studies were included in the final synthesis. The studies were examined according to system type, interaction characteristics, and biomechanical outcomes. The findings indicate a predominance of wearable architectures and inertial sensing technologies in the literature on electronic systems for pediatric gait monitoring. However, HMI modalities were rarely described, and most systems functioned primarily as passive data acquisition tools. Biomechanical outcomes focused mainly on motion-derived parameters, whereas region-specific plantar-load distribution was infrequently assessed, and no studies reported the use of force-sensitive resistors for zonal pressure monitoring. These findings suggest that future advances may depend on integrative approaches that combine multimodal sensing, interaction mechanisms, and functional load characterization.

## 1. Introduction

Gait analysis is a core component of pediatric neuromotor assessment, providing objective indicators of postural control, intersegmental coordination, and locomotor function [1,2]. Electronic monitoring technologies enable the early identification of functional deviations and the quantitative characterization of biomechanical patterns in clinical and developmental contexts [3,4]. Conventional clinical evaluation, however, provides limited quantitative data [5], and traditional laboratory-based approaches—such as optical motion capture, force platforms, and instrumented walkways—require specialized infrastructure that confines assessment to controlled settings and restricts accessibility for continuous or real-world use [6,7].

Wearable IMU-based systems have emerged as portable alternatives that integrate accelerometers, gyroscopes, and magnetometers to capture kinematic signals during natural locomotion [8,9,10]. Combined with machine learning, these systems support automated gait-pattern classification in pediatric populations [11]. Concurrently, robotic and assistive technologies have expanded monitoring capabilities. Inertial approaches have demonstrated feasibility for estimating temporal gait parameters under daily-life conditions in children with cerebral palsy [12], while robot-assisted therapy is increasingly evaluated using quantitative outcomes such as speed, cadence, and walking distance [13].

Despite this progress, substantial heterogeneity in device configurations, outcome metrics, and reporting practices limits cross-study comparability and evidence synthesis [14,15]. Feedback mechanisms such as closed-loop vibrotactile biofeedback remain primarily validated in adult populations, with limited evidence supporting their scalability or clinical integration in pediatric contexts [16]. A structured synthesis examining sensing architectures, HMI mechanisms, and biomechanical outcomes across pediatric gait-monitoring systems is therefore needed.

Accordingly, this review systematically synthesizes peer-reviewed evidence published between 2015 and 2025 on electronic systems for monitoring pediatric gait biomechanics, with emphasis on embedded sensing architectures, HMI mechanisms, and reported biomechanical outcomes. Following PRISMA guidelines and the PROSPERO-registered protocol (CRD420251230372), the review adopts a quantitative descriptive synthesis to characterize technological configurations, interaction modalities, and outcome-reporting patterns. The review is guided by the following research questions:

- RQ1: What types of electronic systems are used to monitor pediatric gait biomechanics, and what are their main technological characteristics?
- RQ2: How are human–machine interface (HMI) modalities defined or omitted in these systems, and how are usability and feedback characteristics described in pediatric contexts?
- RQ3: What biomechanical outcomes are typically measured in pediatric gait monitoring using these systems, and how are they reported?

In this review, the term “used” refers to electronic systems reported in the peer-reviewed literature on pediatric gait biomechanics rather than to the frequency of their adoption in routine clinical gait laboratory practice. These research questions are conceptually grounded in the theoretical framework developed in Section 2, which addresses gait and gait biomechanics, instrumented and embedded systems for pediatric gait assessment, and human–machine interfaces in pediatric gait assessment.

## 2. Theoretical Framework

### 2.1. Gait and Gait Biomechanics

Human gait is a cyclic locomotor pattern in which alternating stance (~60%) and swing (~40%) phases produce forward progression through coordinated limb and trunk motion [17,18]. Clinical quantification relies on three complementary domains: spatiotemporal parameters (speed, cadence, stride length, and phase durations), which are well characterized by IMU-based normative datasets in typically developing children [19]; joint kinematics, which describe angular trajectories throughout the gait cycle; and kinetics, which capture the force-related determinants of support and propulsion [20]. Plantar-loading analysis provides an additional functional dimension by quantifying the spatial distribution of foot–ground contact forces, with pedobarographic approaches enabling pressure-pattern classification relevant to pediatric conditions, including flatfoot [21,22].

These domains are collectively essential in pediatric gait assessment because clinically distinct presentations—including typical development, idiopathic toe walking, and cerebral palsy—require precise and consistently reported descriptors for valid comparison and interpretation [23,24]. IMU-based wearable systems have demonstrated feasibility for objective quantification across these populations [25,26], supporting gait-event identification in idiopathic toe walking [27] and enabling gait-phase recognition through sensor fusion, plantar-pressure measurements, and deep learning pipelines in cerebral palsy [28]. Markerless motion capture represents a complementary laboratory-grade approach [29]. Together, these technologies establish the biomechanical and instrumental foundation from which embedded electronic monitoring architectures are developed.

### 2.2. Instrumented and Embedded Systems for Pediatric Gait Assessment

Wearable electronic systems integrate sensing units, embedded processors, wireless modules, and power-management stages into compact architectures for continuous biomechanical monitoring beyond laboratory environments [30,31], thereby forming acquisition nodes within rehabilitation-monitoring ecosystems [32]. Insole-based configurations are particularly relevant because they directly instrument the foot–ground interface. Smart insoles embed pressure-sensitive and flex-responsive transducers into footwear-compatible multilayer structures with wireless transmission [33,34]. This design space has been systematically documented across prototype configurations [35] and extended to dynamic gait applications through in-shoe plantar-pressure sensing [36]. Additional architectures include flexible strain sensors that conform to body-segment geometry [37], instrumented footwear combining inertial and distance transducers with microcontrollers [38], flexible wearable nodes for real-time kinematic acquisition [39], self-powered insoles that transduce foot–ground mechanical energy into electrical signals [40], and smartphone-embedded inertial sensors that enable portable gait-speed estimation without dedicated hardware [41]. Collectively, these configurations define a multimodal embedded ecosystem spanning clinical and real-world monitoring contexts.

### 2.3. Human–Machine Interfaces in Pediatric Gait Assessment

HMI encompasses the mechanisms by which instrumented systems translate biomechanical data into perceptible, interpretable, and actionable outputs, including dashboards, clinical reports, calibration interfaces, and real-time delivery channels [42,43]. The distinction between assessment and rehabilitation is functionally significant. In assessment contexts, HMI structures outputs for clinical workflows through software analysis, visualization, and structured reporting [44,45], thereby supporting gait-presentation differentiation and treatment evaluation in pediatric populations [46]. Within this construct, biofeedback is defined as the active closed-loop HMI subset in which performance-related information is returned to the user during or after movement to modulate motor behavior [47]. This review adopts the broader HMI framework to encompass the full architectural spectrum, from passive sensing-and-storage designs to systems incorporating haptic, audio, and visual actuators with embedded real-time control logic. In rehabilitation contexts, biofeedback modalities—vibrotactile, visual, and auditory—function as active therapeutic components that facilitate motor correction and learning. This role is further extended through sensor–intelligence integration, which enables adaptive feedback architectures [48].

Despite this dual relevance, HMI mechanisms remain inconsistently incorporated and insufficiently reported in electronic systems for pediatric gait monitoring, with most studies focusing primarily on sensing performance and biomechanical outcomes. To address these limitations in assessment usability and rehabilitation functionality, this review systematically examines sensing architectures (RQ1), HMI and biofeedback characteristics (RQ2), and biomechanical outcomes (RQ3).

## 3. Materials and Methods

This systematic review was conducted in accordance with the PROSPERO protocol (CRD420251230372) and reported following the Preferred Reporting Items for Systematic Reviews and Meta-Analyses (PRISMA 2020) guidelines [49]. The methodological framework was designed to systematically identify, classify, and synthesize evidence on electronic systems applied to the monitoring of pediatric gait biomechanics, with particular emphasis on embedded technologies and HMI.

Although the review primarily focused on embedded technologies and HMI, its scope also included static diagnostic electronic systems and offline monitoring approaches, as defined in the PROSPERO protocol. This broader scope was adopted to identify technological gaps and underexplored areas in pediatric gait biomechanics monitoring. A descriptive quantitative approach was adopted because the primary objective of the review was to characterize the frequency and distribution of system types, embedded monitoring technologies, interaction modalities, and biomechanical outcomes reported across studies. A meta-analysis was not performed because of the heterogeneity in study designs, sensing architectures, clinical contexts, and reporting formats. Accordingly, the findings were integrated through a structured narrative synthesis supported by descriptive quantitative reporting, in accordance with the research questions.

### 3.1. Data Sources and Search Strategy

A systematic literature search was conducted in ScienceDirect, Scopus, and PubMed, which were selected for their complementary coverage of engineering, biomedical technology, and pediatric rehabilitation research. The search included studies published between 1 January 2015 and 1 December 2025, in accordance with the PROSPERO protocol. Only peer-reviewed journal articles published in English were considered to ensure methodological consistency and full-text accessibility.

The search strategies were adapted to the syntax of each database and are presented in Table 1. These strategies combined terms related to pediatric gait analysis, embedded monitoring systems, wearable sensing technologies, inertial sensors, plantar-pressure devices, and human–machine interaction. Boolean operators (AND/OR), truncation (*), and phrase searching using quotation marks were applied as supported by each database interface. The search strings were further adapted according to the syntactic capabilities of each database. Scopus allowed field-specific queries using TITLE-ABS-KEY operators with complex Boolean nesting, whereas ScienceDirect and PubMed required simplified search strings compatible with their native interfaces while preserving the same conceptual search logic across platforms. Publication period and document-type restrictions were applied through the database platforms when available. Accordingly, the search strategy was intentionally designed to prioritize electronic and embedded monitoring systems, including wearable sensing technologies, in line with the technological scope of the review. Therefore, the results should not be interpreted as a comprehensive comparison between wearable systems and all conventional gait-analysis modalities, such as optical or three-dimensional motion capture systems. Similarly, electromyography (EMG) was not used as a primary search term because the review focused primarily on electronic systems for biomechanical gait monitoring, including sensing architectures, wearable technologies, plantar-pressure assessment, and human–machine interaction. However, studies integrating EMG into electronic gait-monitoring systems were eligible when they met the predefined inclusion criteria.

### 3.2. Eligibility Criteria

Studies were selected according to predefined inclusion and exclusion criteria established in the PROSPERO protocol and structured using PICOT-based eligibility criteria. The review focused on non-randomized empirical studies involving pediatric populations and electronic systems for gait assessment with quantitative biomechanical outputs. Language restrictions and the potential implications of excluding gray literature are further acknowledged in the Section 7. Detailed inclusion and exclusion criteria are summarized in Table 2.

### 3.3. Study Selection Process

The study selection process followed the PRISMA 2020 recommendations and comprised three sequential phases: identification, screening, and eligibility assessment, as outlined in the registered PROSPERO protocol (CRD420251230372). During the identification phase, a total of 2619 records were retrieved from ScienceDirect (*n* = 1893), Scopus (*n* = 660), and PubMed (*n* = 66) and imported into the Rayyan online platform (Rayyan Systems Inc., Cambridge, MA, USA) for record organization and screening management. After automatic duplicate detection followed by manual verification, 77 duplicate records were removed, leaving 2542 records for title and abstract screening.

In the screening phase, titles and abstracts were evaluated using Rayyan. This was conducted by one reviewer and independently checked by a second reviewer, in accordance with the PROSPERO protocol. At this stage, 1469 records unrelated to pediatric gait biomechanics or not involving electronic monitoring systems were excluded, and 1073 reports were retained for retrieval.

**Figure 1:**
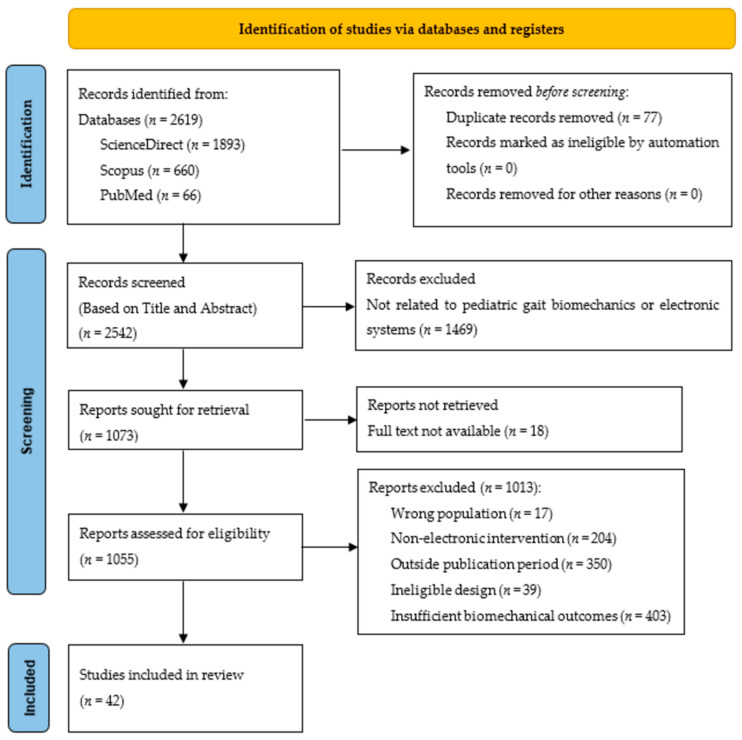
PRISMA 2020 flow diagram of the study selection process prior to methodological quality assessment. Note: The 42 studies retained after eligibility assessment were subsequently subjected to methodological quality appraisal, as described in Section 3.5, to determine the final studies included in the qualitative synthesis.

During the eligibility phase, full-text articles were retrieved for potentially relevant records. Eighteen reports were excluded because the full text was inaccessible, leaving 1055 reports for eligibility assessment. Full-text eligibility was independently assessed by two reviewers according to the predefined criteria, and reasons for exclusion were systematically documented. Disagreements were resolved through discussion and consensus. A total of 42 studies met the eligibility criteria and were included in the review. Figure 1 presents the PRISMA flow diagram of the study selection process up to the inclusion of these 42 studies.

### 3.4. Data Extraction and Synthesis Strategy

Data extraction was performed using a standardized template developed in accordance with the objectives and research questions of the review. The extracted variables were selected to support the classification of electronic monitoring systems and the descriptive synthesis of pediatric gait biomechanical outcomes.

Specifically, the extracted information included basic study characteristics, technological system features, human–machine interaction modalities, and the main reported biomechanical outcomes. Given the variability in sensing configurations, study purposes, and reporting approaches across the included studies, the findings were synthesized using a structured narrative approach supported by descriptive quantitative summaries. This synthesis included frequency-based categorization of technologies, interaction modalities, and biomechanical outcomes to support the identification of underexplored areas and methodological gaps across static and wearable electronic monitoring systems.

As an auxiliary step during data extraction, the SciSpace online platform (PubGenius Inc., Milpitas, CA, USA) was used solely to organize the included articles in relation to RQ1, RQ2, and RQ3. Studies were considered eligible for each synthesis according to their reported characteristics and alignment with the predefined research questions. Specifically, studies were grouped for synthesis based on system type and embedded technology (RQ1), human–machine interaction characteristics (RQ2), and reported biomechanical outcomes and reporting formats (RQ3). No statistical data conversions or imputations were performed. This decision was consistent with the descriptive, non-meta-analytic scope of the review, as defined in the PROSPERO protocol. Given the substantial variability in sensing architectures, clinical populations, assessment contexts, and reporting formats across studies, cross-study normalization could have imposed artificial uniformity and distorted the characterization of technological diversity. Reported measures were therefore retained as presented in the original studies. The results of individual studies and the descriptive syntheses were presented using structured tables and summary figures. Included studies were tabulated according to their characteristics, technological features, interaction modalities, and biomechanical outcomes, whereas frequency-based distributions were displayed through summary figures. Table 3 presents the data-extraction framework aligned with the research questions.

Following data extraction, the methodological quality and risk of bias of the included studies were assessed to support the interpretation and reliability of the synthesized evidence, as described in the following section.

### 3.5. Methodological Quality Assessment and Risk of Bias

Methodological quality and risk of bias were assessed using the Critical Appraisal Skills Program (CASP) Cohort Study Checklist [50], adapted by the authors for non-randomized empirical studies involving wearable, embedded, and static electronic monitoring systems for pediatric gait biomechanics assessment. The adapted framework evaluated key methodological domains, including clarity of study objectives, recruitment strategy, measurement validity, control of confounding factors, completeness of outcome reporting, and applicability of findings, consistent with broader applications of methodological quality assessment tools in evidence synthesis [51].

To ensure suitability for the technological scope of this review, the CASP-based tool was operationalized into 10 appraisal domains covering electronic system description, biomechanical outcome reporting, human–machine interaction characterization, pediatric applicability, methodological design clarity, and real-world implementation potential. Each domain was scored using a 5-point rating scale (1 = very poor; 5 = excellent), with higher scores indicating greater methodological rigor and reporting transparency.

The overall methodological quality score ranged from 10 to 50 points. Studies were classified using author-defined thresholds based on the total score distribution and the need to distinguish studies with acceptable, intermediate, and insufficient methodological quality. Scores ≥ 35 were considered low risk of bias and were included; scores of 30–34 were considered moderate risk of bias and were included with caution; and scores of <30 were considered high risk of bias and were excluded from the synthesis.

Studies categorized as high risk of bias were excluded from the final synthesis. Accordingly, eight studies were excluded following quality assessment. Table 4 presents the adapted CASP-based risk-of-bias criteria for pediatric gait biomechanics studies.

To improve transparency, detailed scoring results for each included study are provided in the Supplementary Materials (Supplementary Materials S1: Risk-of-bias scoring for included studies). For studies classified as moderate or high risk of bias, an additional explanatory note was included to justify the classification and clarify the specific methodological limitations.

Inter-rater agreement was evaluated using Cohen’s kappa coefficient [52] on a randomly selected subsample of eight studies (representing 23.5% of the included studies), independently assessed by an external reviewer. Random selection was performed using a random number generator to reduce selection bias and improve representativeness. This proportion was considered methodologically appropriate, as inter-rater reliability may be assessed using a subset of studies when full-sample appraisal is resource-intensive or time-consuming [53].

The resulting kappa value (κ = 0.789) indicated moderate-to-strong agreement according to commonly used interpretation benchmarks [52]. Statistical analysis was performed using IBM SPSS Statistics for Windows, Version 25.0 (IBM Corp., Armonk, NY, USA). The paired classification dataset used for the reliability assessment and the corresponding SPSS output are provided in the Supplementary Materials (Supplementary Materials S2: Inter-rater reliability dataset and IBM SPSS Statistics Version 25.0 output for Cohen’s kappa analysis).

Studies classified as high risk of bias were excluded from the primary synthesis to prioritize methodologically robust evidence, whereas studies with moderate risk of bias were retained and interpreted with explicit methodological caution in the narrative synthesis. Accordingly, the Section 4 presents the characteristics and synthesized findings of the studies retained after methodological quality assessment.

## 4. Results

A total of 34 studies were included in the final synthesis following full-text screening and methodological appraisal, as illustrated in the PRISMA flow diagram (Figure 1). The included publications span 2015–2025, with a marked predominance in the most recent five-year period. Specifically, 25 studies (73.53%) were published between 2020 and 2025, consistent with the increasing integration of embedded electronic systems in pediatric gait monitoring and the progressive technological maturation of wearable and multimodal platforms.

Geographically, the studies exhibit a heterogeneous international distribution. The United States (*n* = 9) and Italy (*n* = 6) together account for 44.12% of the included studies, reflecting a concentration of research activity within established biomedical engineering and clinical gait-analysis centers. Additional contributions originated from China (*n* = 2), Turkey (*n* = 2), India (*n* = 2), and Republic of Korea (*n* = 2), while the United Kingdom, Spain, France, Japan, Finland, New Zealand, Switzerland, Canada, the Netherlands, and Australia each contributed one study. Overall, this distribution indicates a predominance of high-income countries with specialized technological infrastructures, whereas representation from low- and middle-income regions remains limited.

Table 5 provides an overview of the included studies, detailing the year of publication and country of origin. This serves as a reference framework for the structured synthesis of system characteristics, interaction modalities, and biomechanical outcomes presented in the following sections.

To support structured synthesis and cross-study readability, the included studies were organized into three domain-specific classification tables: Table 6 (electronic system types and embedded technologies), Table 7 (human–machine interaction modalities and operational modes), and Table 8 (biomechanical outcomes and reporting formats). Together, these tables constitute the classification framework applied throughout the synthesis.

### 4.1. Electronic Systems and Embedded Technological Characteristics (RQ1)

In the 34 included studies, electronic systems for monitoring pediatric gait biomechanics were classified according to system type and embedded technology. Wearable systems were the most frequently reported configuration, appearing in 21 studies (61.8%). These systems consisted of body-worn sensing units designed to acquire movement signals in ambulatory settings. Laboratory systems were identified in 10 studies (29.4%) and were based on externally installed sensing infrastructures in controlled environments. Instrumented insoles, integrating sensing components into footwear structures, were reported in two studies (5.9%). Ambient vibration detection systems were described in one study (2.9%), employing environmental sensing mechanisms to detect gait-related signals.

Regarding embedded technologies, inertial measurement units (IMUs) were the most commonly reported component, appearing in 18 studies (52.9%). Multimodal sensing configurations were identified in eight studies (23.5%), integrating combinations of sensing units such as IMUs with plantar-pressure sensors, camera-based systems, electromyography modules, or vibration-sensing components within a single monitoring architecture. Camera-based technologies were reported in four studies (11.8%). Smartphone-based sensing modules appeared in two studies (5.9%), while geophone sensors and pressure platforms were each reported in one study (2.9%). Table 6 presents the categorization of system types and associated embedded technologies identified across the included studies.

While system type describes the operational context of the monitoring solution and embedded technology identifies the sensing components employed, processing architecture defines how the acquired data are handled within the system.

**Figure 2:**
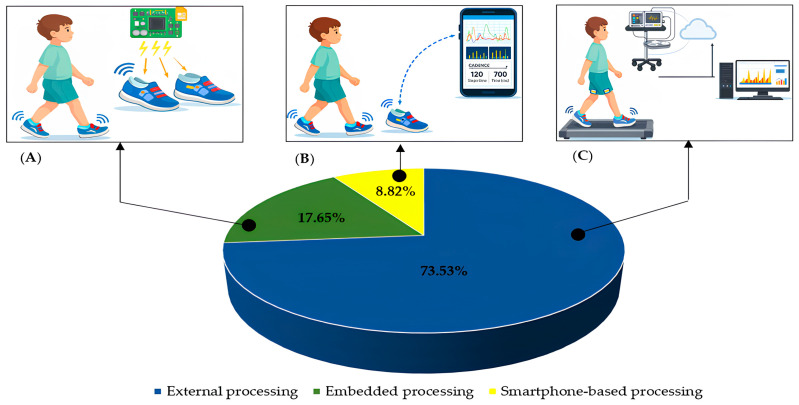
Distribution of processing architectures across the included studies. (**A**) Embedded processing, in which signal handling occurs locally within the monitoring device. (**B**) Smartphone-based processing, in which data acquisition and computation are performed using mobile hardware. (**C**) External processing, in which sensing units transmit data to separate computing environments for analysis.

The distribution of processing architectures across the included studies is presented in Figure 2. External processing was the most frequently reported configuration, appearing in 25 studies (73.53%) [56,57,58,60,61,62,63,64,65,66,69,70,71,72,73,74,75,76,78,80,81,84,85,86], where sensing units transmitted data to separate computing environments for subsequent analysis. Embedded processing architectures were identified in six studies (17.65%) [54,68,77,79,83,87], enabling localized data handling within the monitoring device. Smartphone-based processing was reported in three studies (8.82%) [55,59,67], using mobile hardware for signal acquisition and computation.

Representative conceptual examples of these architectures are illustrated in Figure 2A–C, corresponding to the three identified processing strategies.

Beyond the processing architecture of the monitoring systems, several studies also described how users interacted with these technologies during gait-monitoring or training tasks. The following subsection examines the human–machine interaction characteristics reported across the included studies.

### 4.2. Human–Machine Interaction Modalities and Interface Characteristics (RQ2)

Human–machine interaction characteristics were examined across the included studies according to interaction modality and operational mode. No explicit human–machine interface (HMI) was described in 30 studies (88.24%). Active interaction modalities were reported in four studies (11.76%), where users received feedback during gait-monitoring or training tasks. Operational mode was not reported in 30 studies (88.24%). An online operational mode was identified in four studies (11.76%), where feedback was delivered during task execution. Table 7 summarizes the interaction modalities and operational modes identified across the included studies.

**Figure 3:**
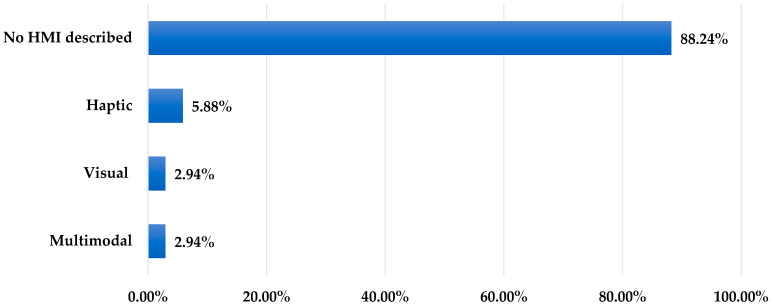
Distribution of feedback modalities among the included studies (*n* = 34). The majority of studies (88.24%, *n* = 30) did not describe any active human–machine interaction (HMI). In the subset incorporating HMI (*n* = 4), haptic feedback was the most common (5.88%, *n* = 2), followed by visual (2.94%, *n* = 1) and multimodal (2.94%, *n* = 1) feedback.

Given that the majority of included studies (88.24%, *n* = 30) included no description of HMI, the following analysis focuses on the subset of studies that incorporated active interaction modalities. Within this subset, three feedback modalities were identified: haptic, visual, and multimodal. Haptic feedback was the most frequently reported modality, whereas visual and multimodal feedback were each identified in one study. The distribution of feedback modalities across the included studies is presented in Figure 3.

Given that only a minority of studies reported active human–machine interaction, the subsequent analysis focuses on the biomechanical parameters of pediatric gait extracted from the included studies.

### 4.3. Reported Biomechanical Outcomes and Measurement Approaches (RQ3)

In accordance with the predefined PROSPERO-registered protocol, biomechanical outcomes were examined in terms of measurement domains and reporting formats.

The biomechanical outcomes of pediatric gait reported across the included studies were grouped into four domains: spatiotemporal, kinematic, stability, and multimodal. These outcomes were derived from a range of sensing configurations, including inertial measurement units (IMUs), pressure sensors, camera-based systems, and integrated multimodal sensing setups.

Multimodal outcomes were the most frequently reported category, identified in 18 studies (52.94%) [54,56,58,59,61,63,68,69,70,72,74,75,77,79,81,83,84,87]. These outcomes integrated signals from multiple sensing sources and were reported using mean ± SD values, normalized gait-cycle representations, or percentage-based metrics. Spatiotemporal parameters were described in eight studies (23.53%) [55,57,62,64,67,71,85,86], typically reported as mean ± SD values (e.g., gait speed, stride length, cadence) or percentage-based measures reflecting gait-phase distribution. Kinematic parameters were reported in four studies (11.76%) [65,66,73,82], most commonly expressed as normalized gait-cycle values (e.g., joint angular profiles across the gait cycle) or proportional metrics. Stability-related parameters were also identified in four studies (11.76%) [60,76,78,80], reported as mean ± SD values (e.g., center of mass sway) or raw measurements reflecting balance deviations.

The outcome categories, reporting formats, and corresponding parameter descriptions are summarized in Table 8.

The observed distribution of biomechanical outcomes and reporting approaches provides a descriptive overview of the measurement focus across the included studies. These findings form the basis for the subsequent discussion, which examines their implications for sensor-based pediatric gait monitoring and the integration of embedded technologies.

## 5. Discussion

### 5.1. Wearable Architectures and IMU-Based Sensing in Pediatric Gait Monitoring

The findings of RQ1 indicate a predominance of wearable architectures and inertial sensing technologies in the reviewed literature, with wearable systems representing 61.8% of the included studies and IMU-based sensing appearing in 52.9%. This distribution reflects growing research interest in portable electronic systems for pediatric gait monitoring. However, recent clinical standards and surveys indicate that the routine adoption of IMUs in clinical gait-analysis laboratories remains relatively limited. One European survey reported their use in approximately 10% of centers, where they were frequently classified as optional equipment [88,89,90]. Accordingly, this review focuses on the technological characteristics, embedded architectures, and human–machine interaction mechanisms of these systems as reported in the research literature rather than on their level of adoption in routine clinical practice.

Traditionally, gait analysis has relied on optical motion capture systems that provide high measurement accuracy but remain constrained by cost, setup complexity, and environmental requirements [91]. These limitations have encouraged the adoption of wearable sensing solutions that support gait assessment beyond controlled laboratory contexts. The predominance of wearable systems identified in the present review reflects broader patterns in gait analysis, in which inertial measurement units (IMUs) have been widely used in real-time gait detection and monitoring frameworks and are commonly positioned on lower-limb segments such as the shank or foot [92]. Their portability and capacity to capture spatiotemporal movement parameters make them practical tools for extending biomechanical analysis into daily-life environments.

Their integration into pediatric clinical contexts has also been supported by feasibility studies showing that IMU-based wearable systems can provide reliable measurements during standardized functional tests while remaining usable for clinicians and patients [93]. Consistent with this, systematic evidence in pediatric populations shows that wearable inertial sensors may support the objective quantification of spatiotemporal gait variables in children, although the limited number of studies and the heterogeneity of clinical conditions still preclude strong recommendations regarding optimal equipment and sensor placement [94].

The multimodal sensing configurations identified in several studies also suggest an emerging trend toward combining IMUs with other sensing modalities to improve signal robustness and contextual interpretation, for example, through integration with insole pressure sensors for ground-truth validation. Despite this shift toward wearable paradigms, laboratory-based systems remain relevant, particularly for validation and benchmarking. Overall, the observed technological distribution suggests a complementary ecosystem in which wearable IMU-based systems support scalable and ecologically valid monitoring, while traditional infrastructures continue to provide high-fidelity biomechanical reference standards. From a clinical application perspective, the wearable and embedded gait-monitoring systems characterized in this review extend beyond data capture to support decision-making in pediatric rehabilitation. By providing objective and repeated assessment of spatiotemporal, kinematic, and load-related parameters in real-world or ambulatory contexts, these systems may assist clinicians in monitoring longitudinal functional changes, detecting gait deviations that require therapeutic adjustment, evaluating responses to orthotic or rehabilitation interventions, and supporting individualized follow-up planning. However, the realization of this clinical utility depends on the interpretability of the extracted parameters, the reliability of the sensing architecture, and the extent to which system outputs are translated into actionable information for clinicians and therapists—dimensions that directly connect sensing architecture to the interaction and output design examined in the following sections. Accordingly, the value of these architectures depends not only on sensing performance but also on how the resulting information is communicated within user and clinical workflows, leading directly to the HMI-related findings addressed in RQ2.

### 5.2. Human–Machine Interaction in Pediatric Gait-Monitoring Systems

The findings of RQ2 reveal a limited presence of explicit HMI components in electronic systems designed for pediatric gait monitoring. In 88.24% of the included studies, no interaction modality was described, and system operational modes were similarly unreported, indicating that most systems remain oriented toward data acquisition rather than user engagement. This pattern suggests that, despite the expansion of wearable sensing architectures identified in RQ1, their translation into interactive clinical or rehabilitation workflows remains limited.

The small proportion of studies reporting active interaction modalities (11.76%) and online operational modes (11.76%) indicates that real-time feedback remains the exception rather than a standard design feature. When present, interaction-oriented approaches demonstrate clinical feasibility and highlight the value of feedback for task adaptation during gait-related activities, supporting a shift from passive monitoring toward intervention-oriented systems [95]. Interaction may also be implemented through device-assisted modalities rather than conventional graphical interfaces. For instance, pediatric robotic gait systems operationalize interaction through control schemes and assistance modes that require coordinated user–device–therapist engagement, thereby directly shaping usability and therapeutic integration [96].

The HMI-related interaction approaches identified in this review can be classified into three functional categories. Feedback-based interaction was observed in all four studies reporting active HMI, delivering performance-related information through haptic [68,83], visual [82], or multimodal channels [79] to support real-time motor adjustment during gait tasks. Assistive interaction—characterized by physical or control-mediated user–device engagement rather than informational feedback—was observed in robotic exoskeleton systems included in this review [54,77], although these studies did not explicitly describe HMI as a distinct interface component. Adaptive interaction, defined as autonomous system adjustment based on real-time user performance analysis, was not identified in any included study. This absence highlights a key design opportunity: intelligent, user-responsive HMI architectures remain an underexplored yet clinically relevant direction for future pediatric gait-monitoring systems.

From a translational perspective, acceptance and tolerability remain essential for real-world implementation. Evidence from pediatric accelerometric monitoring shows that children can accommodate wearable devices in daily-life contexts when systems are unobtrusive and do not disrupt routine activities, underscoring the relevance of user-centered constraints beyond sensor accuracy alone [97]. However, the limited number of studies explicitly reporting interaction mechanisms suggests that many monitoring platforms may not yet be designed with sustained engagement, clinical interpretability, or feedback-driven behavior change as primary objectives.

Overall, the evidence shows a clear gap between sensing capability and interaction functionality: while pediatric gait technologies increasingly support ambulatory data capture, the limited inclusion—and limited reporting—of interactive feedback and operational modes may constrain clinical adoption and reduce translational impact.

Although RQ2 shows that most pediatric gait-monitoring systems remain largely non-interactive, the clinical value of any monitoring or feedback-enabled technology ultimately depends on the biomechanical validity, consistency, and interpretability of the outputs it produces. The next section therefore examines the quantitative biomechanical outcomes reported across studies, focusing on the types of gait parameters measured and their relevance for clinical evaluation and rehabilitation decision-making (RQ3).

### 5.3. Biomechanical Outcomes and Plantar-Pressure Assessment in Pediatric Gait Monitoring

The results of RQ3 indicate that pediatric gait monitoring remains predominantly structured around multimodal and motion-derived outcome domains, despite the increasing adoption of integrated sensing approaches. Multimodal outcomes were the most frequently reported category (52.94%), suggesting that integration has mainly occurred across motion-derived variables rather than functional load-distribution metrics. Heterogeneity in reporting formats—including mean ± SD values, normalized gait-cycle profiles, and percentage-based measures—may further limit cross-study comparability.

Consistent with this pattern, IMU-based analyses continue to provide detailed descriptions of joint motion and gait coordination. For example, multi-segment inertial sensing has enabled the identification of altered movement patterns and symmetry deviations in children with autism spectrum disorder, supporting a more refined characterization of gait mechanics in specific developmental conditions [98].

Similarly, advanced instrumented gait analysis has shown the value of biomechanical parameters for clinical interpretation and treatment planning. For example, integrating gait-derived variables into predictive models has improved decision-support capabilities in children with cerebral palsy [99]. Even within these more sophisticated frameworks, however, biomechanical interpretation remains focused largely on kinematic and temporal measurements rather than on distributed load characteristics.

Studies incorporating plantar-pressure assessment further highlight this limitation. Such measurements are typically obtained from platform-based systems that capture global pressure distributions rather than region-specific loading during dynamic gait. None of the included studies reported the use of force-sensitive resistive (FSR) sensors for zonal plantar-pressure measurement, which may limit the characterization of region-specific load distribution during gait. Importantly, plantar loading is not uniform across the foot. Regional variations between the forefoot, midfoot, and rearfoot have been documented in pediatric populations, including in children with sensory impairments such as hearing impairment [100]. These findings underscore that movement descriptors alone may not fully capture how force is distributed during gait, even though this distribution is thought to be related to propulsion efficiency, balance strategies, and localized mechanical stress.

The clinical implications of this gap are relevant for both diagnosis and rehabilitation. Region-specific plantar-pressure patterns provide load-transfer information that cannot be fully captured by kinematic or spatiotemporal parameters alone. In idiopathic toe walking, forefoot-dominant loading profiles may support functional interpretation and clinical decision-making [21]. In pediatric flexible flatfoot, pressure distribution changes may complement structural assessment and support longitudinal monitoring [22]. In neuromotor disorders such as cerebral palsy, regional loading asymmetries may help identify compensatory strategies not fully evident from joint-angle analysis. From a rehabilitation engineering perspective, real-time insole-based pressure sensing could extend biofeedback systems beyond movement trajectories toward load-distribution control, supporting propulsion training, balance adjustment, and orthotic evaluation. The absence of zonal plantar-pressure sensing therefore constitutes a functionally meaningful limitation in current pediatric gait-monitoring technologies.

Taken together, the outcome patterns identified in RQ3 point to a structural gap in current pediatric gait-monitoring technologies. Although motion and timing metrics are extensively captured, the limited assessment of region-specific plantar load may constrain the functional interpretation of gait biomechanics and the identification of compensatory strategies. Integrating distributed sensing approaches—such as FSR elements positioned across key plantar zones—may offer a potential avenue for complementing kinematic and spatiotemporal measures through real-time detection of load-transfer patterns during gait. However, given the absence of FSR-based studies in the current pediatric gait-monitoring literature, feasibility, accuracy, and clinical utility in pediatric populations require empirical validation before such integration can be recommended.

## 6. Conclusions

This systematic review synthesizes current developments in electronic systems for pediatric gait monitoring and identifies a clear technological shift toward wearable and multimodal sensing approaches that support objective assessment beyond laboratory settings. Despite this progress, most systems remain centered on motion-derived parameters, whereas interaction capabilities and functional load characterization remain limited. Two structural constraints appear to restrict the translational maturity of current pediatric gait-monitoring technologies. First, HMI remains insufficiently developed, with most systems functioning primarily as passive measurement tools rather than interactive platforms capable of supporting feedback-oriented rehabilitation. Second, biomechanical assessment continues to prioritize spatiotemporal and kinematic descriptors, whereas region-specific plantar-load distribution—relevant to propulsion, balance strategies, and compensatory mechanisms—is rarely captured in the included studies. Taken together, these findings suggest that further progress in pediatric gait monitoring will depend on more integrative approaches that combine user-centered HMI with functionally informative load assessment, rather than on the expansion of motion sensing alone. In this context, distributed plantar-pressure sensing may complement multimodal motion capture by improving the biomechanical completeness and interpretability of gait evaluation in real-world settings. More broadly, electronic systems that integrate sensing, HMI, and load-sensitive monitoring may better support personalized and ecologically valid assessment and rehabilitation strategies. Within the 2015–2025 evidence landscape reviewed, most systems still operate primarily as passive monitoring tools, highlighting the need for more interactive and functionally informed designs. To translate these findings into actionable guidance, future research should prioritize integrated three-layer architectures for pediatric gait monitoring. At the sensing layer, IMUs could be combined with distributed FSR sensors across the forefoot, midfoot, and rearfoot zones to capture both motion and plantar-load characteristics. At the processing layer, microcontroller-based embedded units could support local data fusion, real-time signal classification, and wireless transmission. At the HMI layer, haptic or visual actuators could deliver feedback based on both kinematic and plantar-load parameters. Such multimodal architectures would directly address the key gaps identified in this review—biomechanical completeness, HMI functionality, and functional load characterization—while future studies should validate sensor accuracy, data-fusion reliability, clinical usability, user acceptance, and long-term applicability across diverse pediatric populations.

## 7. Limitations

This review has several limitations that should be considered when interpreting its findings. Although it was conducted in accordance with a predefined PROSPERO-registered protocol and restricted to studies published between 2015 and 2025, the synthesis was limited to peer-reviewed literature reporting quantitative biomechanical outcomes in pediatric populations. As a result, relevant developments described in technical reports, prototype-oriented studies, or gray literature may not have been captured. This restriction may also introduce publication bias, which cannot be fully excluded and was considered during interpretation of the descriptive synthesis. In addition, the English-only search strategy may have excluded relevant evidence published in other languages. Substantial heterogeneity in sensing configurations, outcome domains, and reporting formats—including mean ± SD values, normalized gait-cycle profiles, and percentage-based measures—limited cross-study comparability and precluded quantitative aggregation of results. The limited reporting of human–machine interaction modalities and operational features may also reflect incomplete reporting rather than their true absence, as the review relied on explicitly described system characteristics. Finally, the single-person screening and data-extraction processes, although independently checked by another reviewer, may have introduced residual human error. Despite these constraints, this review provides a structured characterization of current technological trends in electronic systems for pediatric gait monitoring.

## Figures and Tables

**Table 1:** Search equations used in ScienceDirect, Scopus, and PubMed.

Database	Search Equation
Scopus	TITLE-ABS-KEY ((“electronic system*” OR “embedded system*” OR “wearable*” OR “sensor*” OR “inertial measurement unit*” OR “IMU*” OR “force sensing resistor*” OR “FSR sensor*” OR “Arduino” OR “ESP32” OR “Internet of Things” OR “IoT” OR “machine learning” OR “artificial intelligence”) AND (“gait analysis” OR “gait monitoring” OR “biomechanical analysis” OR “plantar pressure” OR “spatiotemporal parameter*” OR “kinematic*” OR “kinetic*” OR “walking pattern*” OR “orthopedic insole*” OR “orthopedic shoe*” OR “orthotic*” OR “custom insole*” OR “foot orthosis” OR “orthopaedic footwear”) AND (“pediatric” OR “child*” OR “children” OR “infant*” OR “toddler*”) AND (“user interface*” OR “mobile application*” OR “app*” OR “dashboard*” OR “human computer interaction” OR “HCI” OR “real time monitoring” OR “visualization”))
ScienceDirect	(“wearable sensor” OR IMU OR “embedded system” OR “electronic gait”) AND (pediatric OR child) AND (gait OR walking OR “biomechanical analysis”)
PubMed	(“wearable sensor” OR IMU OR “embedded system” OR “electronic gait”) AND (pediatric OR child) AND (gait OR walking OR “biomechanical analysis”)

**Table 2:** Inclusion and exclusion criteria.

Inclusion Criteria	Exclusion Criteria
Children aged 0–17 years with typical development or gait, postural, or locomotor abnormalities. Mixed-population studies were permitted if pediatric data were reported separately.	Adults (over 18 years old), animal studies, or studies in which pediatric biomechanical data could not be isolated. Studies without a pediatric age focus were excluded.
Electronic systems for pediatric gait monitoring, including static platforms and dynamic, embedded, or wearable devices with electronic hardware (e.g., sensors and processing units) and HMIs (active/passive; online/offline). Systems were required to generate quantitative biomechanical data.	Non-electronic or observational methods; systems without electronic hardware; technologies designed exclusively for adults; devices without biomechanical measurement capabilities.
Traditional non-electronic methods (observational/manual), when present, were used only for contextual reference and were not a requirement for inclusion.	Studies in which comparisons involved non-electronic technologies without describing electronic systems.
Biomechanical parameters and locomotor deviations reported in pediatric gait (e.g., symmetry, rotation, joint angles, and center of pressure).	Studies without quantitative biomechanical data or gait parameters.
Studies conducted in clinical, rehabilitation, laboratory, or community settings related to pediatric gait assessment, with no restrictions based on country, health system, or economic level.	Non-clinical settings with no relevance to pediatric gait (e.g., industry or sports settings without children).

**Table 3:** Data-extraction framework aligned with the research questions.

Category	Extracted Variable	Description
Study characteristics	Year, country	Basic descriptive information on the included studies.
RQ1	System type, embedded technology	Wearable systems, instrumented insoles, pressure platforms, and microcontroller-based or smartphone-based architectures.
RQ2	Interaction modality, feedback type	Active/passive interaction; online/offline operation; visual, audio, or haptic feedback.
RQ3	Main biomechanical outcomes, reporting format	Spatiotemporal parameters (cadence, step length), plantar pressure (COP, peak pressure); mean ± SD; normalized gait cycle.

**Table 4:** Adapted risk-of-bias criteria for pediatric gait biomechanics studies.

N°	Criterion Aligned with Review Objectives	Evaluation Question	Rating Scale				
			1	2	3	4	5
1	Electronic system used	Does the study clearly describe the type of electronic system employed?					
2	Biomechanical indicators monitored	Does the study detail the biomechanical parameters evaluated?					
3	User interaction mode	Does the study describe the mode of visualization or feedback?					
4	Pediatric applicability	Does the study population correspond to children aged 0–17 years?					
5	Methodological design	Does the study present a clear, replicable, and validated methodological design?					
6	Quality of results reporting	Are the results adequately presented (tables, graphs, analyses)?					
7	Technological relevance and innovation	Does the system represent an advancement over traditional methods?					
8	Real-world applicability	Can the system potentially be implemented outside the laboratory?					
9	Recognition of limitations	Does the study report its limitations or design restrictions?					
10	Alignment with this review’s objectives	Is the study clearly aligned with one or more review question?					

**Table 5:** Characteristics of the 34 studies included in the systematic review and synthesis.

Reference	Title	Year	Country
[54]	Robotic Knee Exoskeletons as Assistive and Gait Training Tools in Spina Bifida: A Pilot Study Showing Clinical Feasibility of Two Control Strategies	2025	USA
[55]	Harnessing Fast Fourier Transform for Rapid Community Travel Distance and Step Estimation in Children with Duchenne Muscular Dystrophy	2025	USA
[56]	Entropy, Irreversibility, and Time-Series Deep Learning of Kinematic and Kinetic Data for Gait Classification in Children with Cerebral Palsy, Idiopathic Toe Walking, and Hereditary Spastic Paraplegia	2025	Spain
[57]	Gait temporal parameters estimation in toddlers using inertial measurement units: A comparison of 15 algorithms	2025	United Kingdom
[58]	Management of cognitive-motor interference in dual-task walking among healthy children aged 7–12 years	2025	France
[59]	The relationship between gait profile and spino-pelvic alignment in patients with adolescent idiopathic scoliosis of Lenke type 1 and 5	2025	Republic of Korea
[60]	Influence of Main Thoracic and Thoracic Kyphosis Morphology on Gait Characteristics in Adolescents with Idiopathic Scoliosis: Gait Analysis Using an Inertial Measurement Unit	2025	Japan
[61]	Long term gait postural characteristics of children with general foot pain using smartphone connected wearable sensors	2025	China
[62]	Ambient floor vibration sensing advances the accessibility of functional gait assessments for children with muscular dystrophies	2024	USA
[63]	3D gait analysis in children using wearable sensors: feasibility of predicting joint kinematics and kinetics with personalized machine learning models and inertial measurement units	2024	New Zealand
[64]	Effects of interval treadmill training on spatiotemporal parameters in children with cerebral palsy: A machine learning approach	2024	USA
[65]	Explainable Deep-Learning-Based Gait Analysis of Hip–Knee Cyclogram for the Prediction of Adolescent Idiopathic Scoliosis Progression	2024	Republic of Korea
[66]	Prediction of joint moments from kinematics using machine learning in children with congenital talipes equino varus and typically developing peers	2024	India
[67]	Gait Event Detection and Travel Distance Using Waist-WornAccelerometers across a Range of Speeds: Automated Approach	2024	USA
[68]	Balance control via tactile biofeedback in children with cerebral palsy	2023	Turkey
[69]	A Supervised Classification of Children with Fragile X Syndrome and Controls Based on Kinematic and sEMG Parameters	2022	Italy
[70]	Gait performance in toddlers born preterm: A sensor based quantitative characterization	2022	Italy
[71]	Validation of shoe-worn Gait Up Physilog^®^5 wearable inertial sensors in adolescents	2022	Australia
[72]	Test–retest reliability and minimal detectable change for measures of wearable gait analysis system (G-Walk) in children with cerebral palsy	2022	Turkey
[73]	A Markerless-based Gait Analysis and Visualization Approach for ASD Children	2021	Malaysia
[74]	Multi-body sensor data fusion to evaluate the hippotherapy for motor ability improvement in children with cerebral palsy	2021	China
[75]	Reliability of single-day walking performance and physical activity measures using inertial sensors in children with cerebral palsy	2021	Switzerland
[76]	The gait is less stable in children with cerebral palsy in normal and dual task gait compared to typically developed peers	2021	Finland
[77]	A Pediatric Knee Exoskeleton With Real-Time Adaptive Control for Overground Walking in Ambulatory Individuals With Cerebral Palsy	2021	USA
[78]	Human motor control: Is a subject-specific quantitative assessment of its multiple characteristics possible? A demonstrative application on children motor development	2020	Italy
[79]	The validity and usability of an eight marker model for avatar-based biofeedback gait training	2019	Netherlands
[80]	Dynamic balance assessment during gait in children with Down and Prader–Willi syndromes using inertial sensors	2019	Italy
[81]	A wearable gait analysis protocol to support the choice of the appropriate ankle-foot orthosis: A comparative assessment in children with Cerebral Palsy	2019	Italy
[82]	Visual kinematic feedback enhances the execution of a novel knee flexion gait pattern in children and adolescents	2019	USA
[83]	Customized Wereable Sensor-Based Insoles for Gait Re-Training in Idiopathic Toe Walkers	2019	USA
[84]	The Pediatric SmartShoe: Wearable Sensor System for Ambulatory Monitoring of Physical Activity and Gait	2018	USA
[85]	Validation of a commercial inertial sensor system for spatiotemporal gait measurements in children	2017	Canada
[86]	Gait parameters in school going children using a marker-less approach	2016	India
[87]	Multiple gait patterns within the same Winters class in children with hemiplegic cerebral palsy	2015	Italy

**Table 6:** Categorization of electronic system types and embedded technologies.

System Type	Embedded Technology	Description	Reference(s)
Wearable	IMU	Portable sensors on limbs or trunk for kinematic signal acquisition with wireless transmission.	[57,60,61,63,65,68,70,71,72,74,75,76,78,80,81,82,85]
	Smartphone	Integrated sensing modules for motion signal acquisition with on-device processing.	[55,67]
	Multimodal	Sensor combinations, including IMUs and additional sensing units.	[54,77]
Laboratory	Camera system	Optical motion capture for joint kinematic tracking in controlled settings.	[66,73,79,86]
	Multimodal	Sensor fusion approaches, including pressure platforms and IMUs.	[56,58,69,87]
	Pressure platform	Force platforms for spatiotemporal signal acquisition during walking tasks.	[64]
	IMU	Inertial sensors integrated within controlled gait-analysis infrastructures.	[59]
Instrumented insole	Multimodal	Embedded insoles for plantar signal acquisition.	[83,84]
Ambient floor vibration sensing	Geophone sensors	Non-contact vibration-sensing systems for gait-related signal detection.	[62]

**Table 7:** Human–machine interaction characteristics identified across the included studies.

Interaction Modality	Operational Mode	Description	Reference(s)
No HMI described	Not reported	No explicit user–system interaction was described in these studies.	[54,55,56,57,58,59,60,61,62,63,64,65,66,67,69,70,71,72,73,74,75,76,77,78,80,81,84,85,86,87]
Active	Online	Haptic feedback delivered through wearable vibration actuators to guide balance control during walking tasks.	[68]
		Multimodal feedback combining visual and auditory cues within an immersive virtual environment for gait training.	[79]
		Visual feedback providing real-time kinematic information to support execution of targeted gait patterns.	[82]
		Haptic feedback delivered through insole-based tactile stimulation to facilitate gait retraining.	[83]

**Table 8:** Categories of biomechanical outcomes reported in the included studies.

Outcome Category	Reporting Format	Percentage	Description	Reference(s)
Multimodal	Mean ± SD	32.35%	Integrated outputs combining multiple sensor signals, including entropy-based classification and vibrotactile-related metrics.	[54,56,58,59,61,68,72,75,77,81,84]
	Normalized cycle	5.88%	Integrated parameters over gait-cycle phases.	[74,79]
	Percentage	14.71%	Relative contributions or classification accuracy of fused data.	[63,69,70,83,87]
Spatiotemporal	Mean ± SD	20.59%	Gait speed, stride length, cadence, stance/swing-phase duration, step time.	[55,57,64,67,71,85,86]
	Percentage	2.94%	Relative proportions of gait phases or symmetry indices.	[62]
Kinematic	Normalized cycle	8.82%	Joint angles (hip, knee, and ankle) expressed as percentage of gait cycle.	[66,73,82]
	Percentage	2.94%	Angular displacements or velocity profiles.	[65]
Stability	Mean ± SD	8.82%	Center of mass sway, trunk stability metrics.	[60,76,80]
	Raw values	2.94%	Direct sway or balance deviation measures.	[78]

## Data Availability

The data supporting the findings of this study are available within the article and its Supplementary Materials. Supplementary Material S1 contains the risk-of-bias scoring for included studies, and Supplementary Material S2 contains the inter-rater reliability dataset and SPSS v25 output for Cohen’s kappa analysis.

## Supplementary Materials

The following supporting information can be downloaded at: https://www.mdpi.com/article/10.3390/s26103164/s1, Supplementary Materials S1: Risk-of-bias scoring for included studies; Supplementary Materials S2: Inter-rater reliability dataset and SPSS v25 output for Cohen’s kappa analysis.

## Abbreviations

The following abbreviations are used in this manuscript:

CASP	Critical Appraisal Skills Programme
COP	Center of Pressure
GRF	Ground Reaction Force
HMI	Human–Machine Interaction
IMU	Inertial Measurement Unit
PRISMA	Preferred Reporting Items for Systematic Reviews and Meta-Analyses
PROSPERO	International Prospective Register of Systematic Reviews
RQ	Research Question
RoB	Risk of Bias
ROM	Range of Motion
SD	Standard Deviation
