# Supplementary material for: Electronic Systems for Monitoring Pediatric Gait Biomechanical Parameters: A Systematic Review of Embedded Technologies and Human–Machine Interfaces
Source: Sensors (Basel). 2026 May 16;26(10):3164. doi: 10.3390/s26103164 (PMC13210630; doi:10.3390/s26103164)
Supplement: Supplementary file 1 [file sensors-26-03164-s001.zip › Supplementary Material S2.pdf]

Supplementary Material S2: Inter-Rater Reliability Dataset and SPSS Output for Cohen's Kappa Analysis

Table S1 presents the paired risk-of-bias classifications assigned independently by both reviewers for the randomly selected subsample (20% of included studies). The table includes total CASP scores and the corresponding categorical risk classification (low, moderate, high) used for agreement analysis.

Table S1. Inter-Rater Reliability Classification Dataset (n = 8)

N°	Reference	Title	Total score Rater 1	Risk category rater 1	Total score rater 2	Risk category rater 2
**1**	(Volkan-Yazici et al., 2022)	Test-retest reliability and minimal detectable change for measures of wearable gait analysis system (G-Walk) in children with cerebral palsy	41	Low risk	39	Low risk
**2**	(Traistaru et al., 2025)	Chronic Implications of Bilateral Foot Pattern Variability in Schoolchildren	29	High risk	28	High risk
**3**	(Antunes et al., 2016)	Different horse's paces during hippotherapy on spatio-temporal parameters of gait in children with bilateral spastic cerebral palsy: A feasibility study	37	Low risk	38	Low risk
**4**	(Li et al., 2021)	A wearable MARG sensor-based method for the assessment of the effectiveness for hippotherapy in CP children	34	Moderate risk	33	Moderate risk
**5**	(Choi et al., 2025)	The relationship between gait profile and spino-pelvic alignment in patients with adolescent idiopathic scoliosis of Lenke type 1 and 5	35	Low risk	32	Moderate risk
**6**	(DeVol et al., 2025)	Effects of interval treadmill training on spatiotemporal parameters in children with cerebral palsy: A machine learning approach	36	Low risk	37	Low risk
**7**	(Li et al., 2021)	Age-related gait development in children with autism spectrum disorder	28	High risk	29	High risk
**8**	(Bisi & Stagni, 2020)	Human motor control: Is a subject-specific quantitative assessment of its multiple characteristics possible? A demonstrative application on children motor development	39	Low risk	40	Low risk

Figure S1 illustrates the SPSS data structure used for the inter-rater agreement calculation, displaying the coded categorical variables for RiskCategory_Rater1 and RiskCategory_Rater2 prior to statistical analysis.

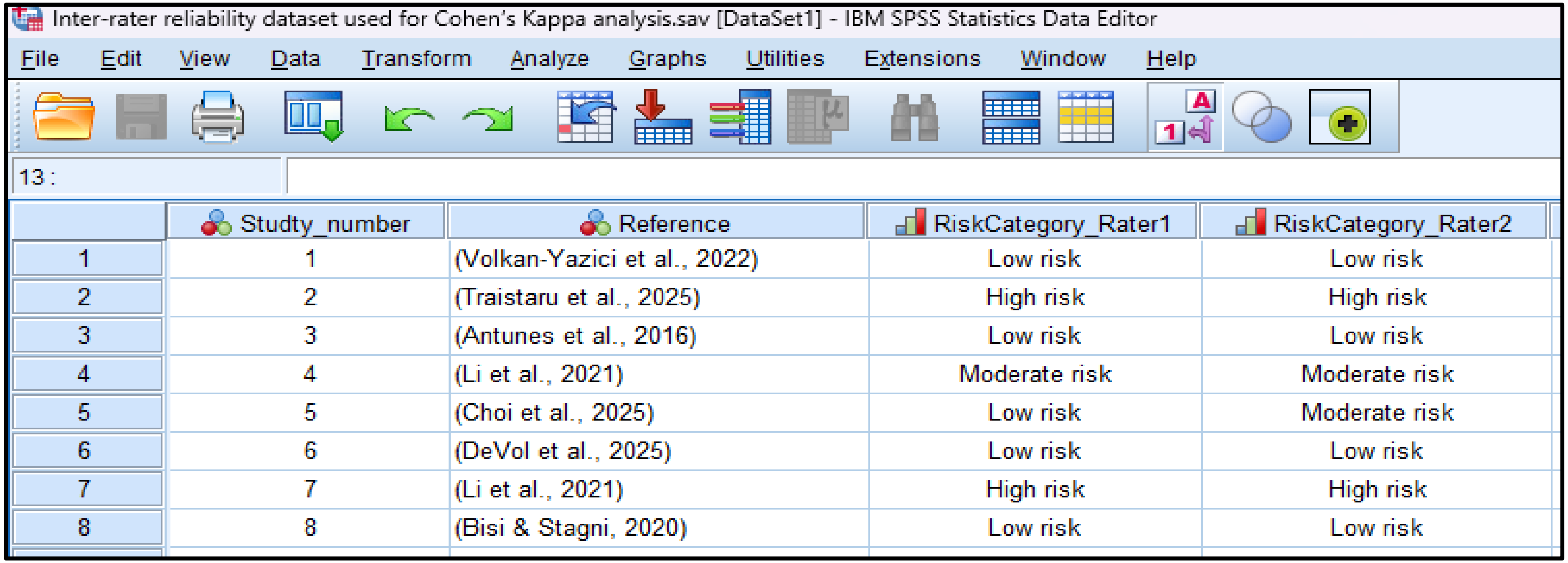

	Studty_number	Reference	RiskCategory_Rater1	RiskCategory_Rater2
1	1	(Volkan-Yazici et al., 2022)	Low risk	Low risk
2	2	(Traistaru et al., 2025)	High risk	High risk
3	3	(Antunes et al., 2016)	Low risk	Low risk
4	4	(Li et al., 2021)	Moderate risk	Moderate risk
5	5	(Choi et al., 2025)	Low risk	Moderate risk
6	6	(DeVol et al., 2025)	Low risk	Low risk
7	7	(Li et al., 2021)	High risk	High risk
8	8	(Bisi & Stagni, 2020)	Low risk	Low risk

Figure S1. Inter-Rater Reliability Dataset in SPSS (Data View)

Figure S2 presents the SPSS crosstabulation matrix and the corresponding Cohen's Kappa statistics, including the agreement distribution across risk categories and the calculated Kappa coefficient (κ = 0.789), standard error, and significance level.

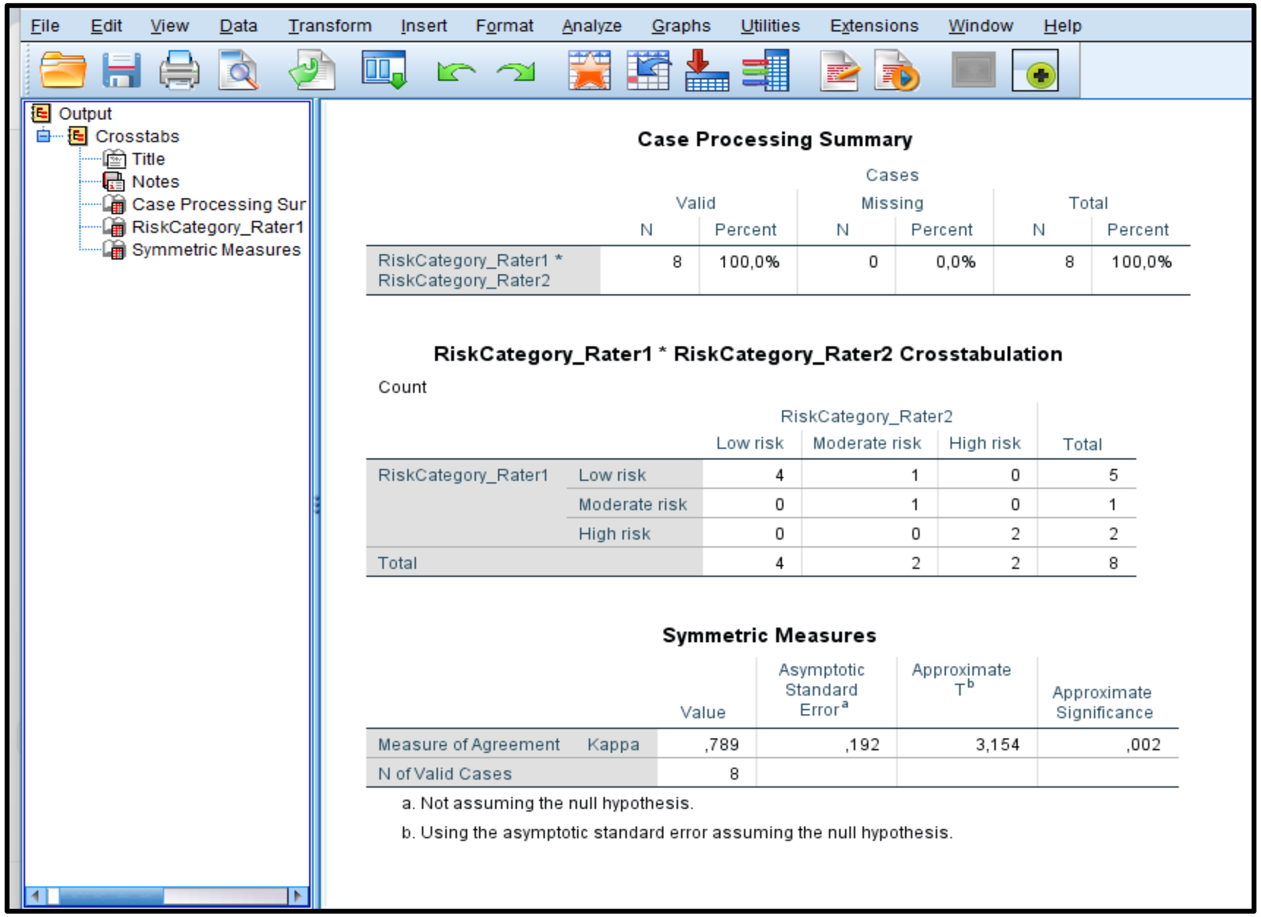

Case Processing Summary

	Cases					
	Valid		Missing		Total	
	N	Percent	N	Percent	N	Percent
RiskCategory_Rater1 * RiskCategory_Rater2	8	100,0%	0	0,0%	8	100,0%

RiskCategory_Rater1 * RiskCategory_Rater2 Crosstabulation

Count

		RiskCategory_Rater2			
		Low risk	Moderate risk	High risk	Total
RiskCategory_Rater1	Low risk	4	1	0	5
	Moderate risk	0	1	0	1
	High risk	0	0	2	2
Total		4	2	2	8

Symmetric Measures

		Value	Asymptotic Standard Error[a]	Approximate T[b]	Approximate Significance
Measure of Agreement	Kappa	,789	,192	3,154	,002
N of Valid Cases		8			

a. Not assuming the null hypothesis.

b. Using the asymptotic standard error assuming the null hypothesis.

Figure S2. SPSS Crosstabulation and Cohen's Kappa Output
